# ContDist: a tool for the analysis of quantitative gene and promoter properties

**DOI:** 10.1186/1471-2105-10-7

**Published:** 2009-01-07

**Authors:** Michael Hackenberg, Gorka Lasso, Rune Matthiesen

**Affiliations:** 1 Bioinformatics Group, CIC bioGUNE, CIBER-HEPAD, Technology Park of Bizkaia, 48160 Derio, Bizkaia, Spain; 2 Functional Genomics Unit, CIC bioGUNE, CIBER-HEPAD, Technology Park of Bizkaia, 48160 Derio, Bizkaia, Spain; 3 Structural Biology Unit, CIC bioGUNE, Technology Park of Bizkaia, 48160 Derio, Bizkaia, Spain; 4 IPATIMUP, Rua Dr. Roberto Frias s/n, 4200-465 PORTO, Portugal

## Abstract

**Background:**

The understanding of how promoter regions regulate gene expression is complicated and far from being fully understood. It is known that histones' regulation of DNA compactness, DNA methylation, transcription factor binding sites and CpG islands play a role in the transcriptional regulation of a gene. Many high-throughput techniques exist nowadays which permit the detection of epigenetic marks and regulatory elements in the promoter regions of thousands of genes. However, so far the subsequent analysis of such experiments (e.g. the resulting gene lists) have been hampered by the fact that currently no tool exists for a detailed analysis of the promoter regions.

**Results:**

We present ContDist, a tool to statistically analyze quantitative gene and promoter properties. The software includes approximately 200 quantitative features of gene and promoter regions for 7 commonly studied species. In contrast to "traditionally" ontological analysis which only works on qualitative data, all the features in the underlying annotation database are quantitative gene and promoter properties.

Utilizing the strong focus on the promoter region of this tool, we show its usefulness in two case studies; the first on differentially methylated promoters and the second on the fundamental differences between housekeeping and tissue specific genes. The two case studies allow both the confirmation of recent findings as well as revealing previously unreported biological relations.

**Conclusion:**

ContDist is a new tool with two important properties: 1) it has a strong focus on the promoter region which is usually disregarded by virtually all ontology tools and 2) it uses quantitative (continuously distributed) features of the genes and its promoter regions which are not available in any other tool. ContDist is available from

## Background

Enrichment/depletion analysis of gene lists derived from high-throughput experiments is nowadays an established and important procedure which helps to analyze and interpret the output of an experiment under a system biology point of view [1]. A textbook example is the differential expression of genes under pathologic conditions like cancer. The differential expressed genes are likely to be important in the development of the pathology and it is therefore important to link them to biological knowledge available in databases. The enrichment or depletion of functional ontologies for these genes gives a valuable overview on the molecular bases of the analyzed pathology.

The first tool developed for this kind of analysis was Onto-Express which used functional annotations from the Gene Ontology [2]. Since then many different tools have been developed like FatiGO+ [3], DAVID [4], the further development of Onto-Express [5] or recently Annotation-Modules [6] (see also [7] for a review and [8] for the gene set enrichment approach for differentially expressed genes). The goal of these methods is to detect gene/protein properties which are significantly over or underrepresented in a user given input list. The exact null distribution for this problem is the hypergeometric distribution [9], and statistical tests like the Fisher exact test can be implemented to calculate the statistical significance of the depletion/enrichment. The dichotomous character of these statistical tests imply directly that just qualitative gene properties can be used as annotations, e.g. those which can be assigned as a label like 'transcription' or 'miR-1'. However, many biologically interesting gene properties may not be qualitative but quantitative, e.g. continuously distributed (or discrete distribution with a high number of different values). An important example is the number of Protein-Protein-Interactions (PPI) in which a gene product is involved. The number of interactions can reach from 1 to hundreds in the case of hub proteins. In such cases, dichotomous statistical tests (like hypergeometric or binomial) cannot be directly applied unless the data is transformed (for example dichotomization into interactors and non-interactors). The discretization of continuous data potentially removes noise but will also suffer the loss of information and the effects of arbitrary classification (number of bins, equal bin frequencies vs. equal bin width etc.). The analysis of the PPI is currently only available in FatiGO+ [3] implementing a parameter free Kolmogorov-Smirnov test.

Apart from the mentioned Protein-Protein-Interactions, many other important quantitative gene properties can be conceived. Examples of continuously distributed quantitative features are those related to sequence evolution such as the Ka/Ks ratio or substitution rates, the sequence composition like the G+C content or the codon bias. Moreover, most of the tools for the analysis of gene lists focus on the gene products and less attention is paid to the promoter regions despite its importance in the regulation of gene expression. Thus ignoring important features like helical deformations (physical DNA properties have been shown to determine nucleosome occupancy and are therefore crucial in the regulation of gene expression [10]), dinucleotides densities, base composition or the degree of overlap with genomic elements like transposable elements or phylogenetically conserved elements [11]. Moreover, current improvements in high-throughput techniques have a higher emphasis on the promoter region allowing now the experimental determination of methylation states, epigenetic marks or RNA polymerase occupancy of thousands of promoters simultaneously. Therefore, to further characterize the resulting genes and its promoters (the genes and promoter which summarize the experiment), a tool which can handle quantitative features and with a strong focus on the promoter regions would be of great importance.

We developed ContDist, a web based tool which analyses and compares user provided gene lists. The novelty of the tool is that all the available features are quantitative annotations which cannot be analyzed in any existing tool. Furthermore, no other tools have a strong focus on the promoter region. Currently, the tool implements approx. 200 different annotations. Several of these annotations are highly relevant in many studies such as Ka/Ks ratio, physical DNA properties and base composition of promoter regions, overlap with genomic elements and gene expression. We demonstrate the usefulness and functionality of this tool, by means of two case studies. The first case study, a comparison of a list of genes with unmethylated promoters with a list of differentially methylated promoters, confirms some recent findings like markedly different CG, CA and TG densities. In both case studies we also identified new significant correlations which are detailed in the "Results and discussion" section.

## Results and discussion

### Algorithm and data flow

**Figure 1:**
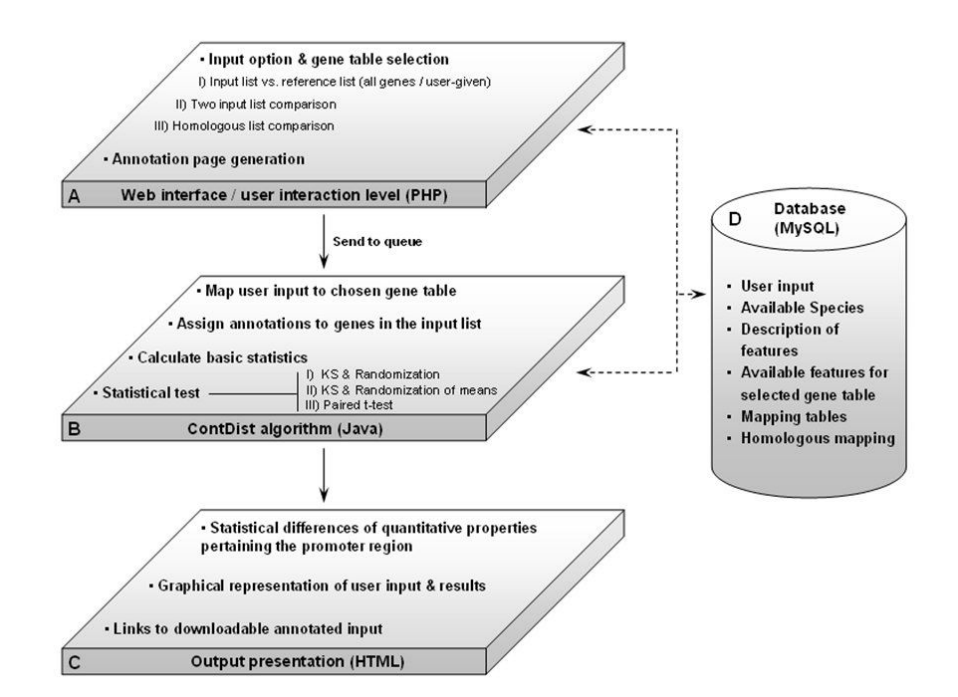
**Outline of data flow**. ContDist is composed of three separate layers along with a MySQL database. The top layer (A) corresponds to the web interface where the user input is handled, the available promoter properties are retrieved from the MySQL database and the information is parsed to the middle layer (B). The middle layer (B) performs all mappings, retrieves the values of the promoter properties to be statistically analyzed, applies the appropriate statistical tests and parses the data to the bottom layer (C). The bottom layer generates a HTML-based output describing the statistical differences detected for the chosen annotations in the input data. Dashed arrows correspond to the communication of the layers with the MySQL database whereas plain arrows correspond to the dataflow between layers.

Three different input options are available (Figure 1): i) comparison of an input gene list to a background reference gene set (the reference set can be all genes in the genome for which information on the analyzed annotation exists, or it can be user defined); ii) comparison of two user provided gene lists; and iii) comparison of an input gene list to the corresponding homologous genes in another specie (the comparison can be done with all the other 6 species in the database). Depending on the input different statistical tests are performed.

### Annotations

The current version of the annotation database holds information on 7 species, human (*Homo sapiens*), mouse (*Mus musculus*), rat (*Ratus norvegus*), fruit fly (*Drosophila melanogaster*), chimpanzee (*Pan troglodites*), zebrafish (*Danio rerio*) and cow (*Bos taurus*). Depending on the species between 180 and 220 annotations are available. These annotations can be clustered into six different categories: i) physical properties of DNA and chromatin, ii) base composition, iii) evolution, iv) general gene/protein properties, v) overlap with genomic elements and vi) gene expression. A short summary of the available annotation features are provided in table 1. A more detailed description of the annotations can be found on .

**Table 1:** Summery of the different quantitative features used by ContDist.

Feature group	Features
Physical properties of DNA and chromatin	Helical deformations, predictions of methylation state
Base composition	G+C content, density of dinucleotides
Evolution	SNPs from dbSNP, and Substitution rates (Ka, Ks, Knr, Knc, Ka/Ks, etc)
General gene/protein properties	PPI, codon bias, gene structure
Overlap with genomic elements	Repetitive elements, PhastCons, CpG islands
Gene expression	Expression values form gene atlas, expression breath, maximum and average expression

Numerous properties are assigned in a genomic context and therefore a classification of the gene and promoter regions is needed. Apart from the "intrinsic" gene regions like exons, introns and untranslated regions, several promoter regions are defined which are described in [6].

#### Physical DNA and chromatin properties

The positioning of the nucleosomes plays an important role in cellular processes like the regulation of gene expression by means of modulating the accessibility of DNA (chromatin state) [12]. There is evidence that the nucleosome formation and/or positioning depends on intrinsic properties of the DNA sequence such as flexibility or natural bending of adjacent base pairs [10,13]. In particular, the repetition of curved DNA motifs positioned at intervals of one turn of the double helix can contribute to DNA curvature and facilitates its wrapping around the histone surface. Therefore the mean values for 6 helical deformations (Twist, Tilt, Roll, Shift, Slide and Rise) were computed in different promoter regions by using the stiffness constants given in [14]. Additional features related to DNA methylation are also implemented as it has been described that the methylation of the 5'cythosine of CpG dinucleotides is related to compact and inaccessible chromatin state [15]. Two predictions on the methylation probabilities and epigenetic states were subsequently considered. Das *et al *[16] predict a methylation probability for each single CpG in the human autosomes of the human genome assembly hg15 (NCBI 33). The coordinates were first mapped to the most recent genome assembly (NCBI 36.1, hg18) using the liftOver tool from UCSC . Next, the mean probability to remain unmethylated was computed for the CpGs located within the promoter regions (for different definitions). Recently, Bock *et al *proposed a method capable of predicting the "CpG island strength" by considering the epigenetic states, histone modifications and chromatin accessibility [17]. Using this prediction it was possible to assign an epigenetic score to each promoter region containing at least one CpG island.

#### Base composition

The base composition category contains basically the GC-content and dinucleotide densities in different gene and promoter regions. The density of methylable dinucleotides (in mammals basically CpG) may be of special interest as it is known that it correlates with the probability to become methylated [18]. On the other site, dinucleotides like TG and CA frequently arise by methylation and posterior mutation of CpGs and therefore the densities of these dinucleotides may also be interesting in evolutionary terms.

#### Evolution

An interesting feature is the Ka/Ks ratio which allows the user to detect if different selective constraints acted on the genes in the input list [19]. The Ka/Ks ratios between different species were extracted out of the homologene.xml file provided by HomoloGene . Furthermore, several other values regarding the nucleotide and amino acid substitution rates were obtained from this file.

#### SNPs

The SNP density in different gene and promoter regions is also available in the annotation database for human, mouse and rat. The SNP information was retrieved from dbSNP126 for human and mouse and version 125 for rat.

#### General gene/protein properties

This category holds some miscellaneous features like the number of Protein-Protein interactions a gene product is involved in or the codon bias. The mortality or lethality of specific protein mutations in Yeast have been shown to correlate with the number of protein-protein interactions (the centrality in protein networks) [20]. Based on this knowledge we extracted the number of protein interactions for every protein based on the information in the Interact database  without distinguishing between the types of interaction. This means that ContDist can be used to test the hypothesis that proteins with many interactions correlate with severe phenotypes. The effective number of codons was calculated by means of Wright's formula [21] as explained in [6].

#### Overlap with genomic elements

The presence of transposable elements in untranslated and promoter regions is believed to affect gene expression through the donation of transcriptional regulatory signals [22]. Therefore, the differential degree of overlap with transposable elements between two gene lists might be an interesting biological feature to be considered. Therefore the coverage of different gene and promoter regions with transposable elements and repeats was computed by using the appropriate data from the UCSC table browser (RepeatMasker prediction). Furthermore, particular CpG island properties were assigned to all genes which have an island overlapping its TSS (Transcription Start Site). In concrete the GC-content, the observed/expected ratio, the CpG density and the length of the islands predicted by *CpGcluster *[23] were assigned.

#### Gene Expression

The percentage of tissues where the gene is expressed, the mean expression over all tissues and the peak expression rate was computed from the gene atlas for human and mouse transcripts [24]. The expression data was downloaded from the UCSC table browser . All probes with lower expression values than 200 units [25] were filtered out and the remaining probes were subsequently averaged over different probes of the same gene.

### IDs and gene lists

For human, mouse and rat the mapping concept introduced in Annotation-Modules  was reused allowing many different identifiers as input for these species. Given the strong focus on the promoter regions of the genes, the available annotations have been calculated mainly for two different gene tables, RefSeq genes [26] and Ensembl genes [27]. For the fruit fly, FlyBase gene tables were considered as well [28].

### Homologous genes

The data from HomoloGene is used to generate the mapping from the input gene identifiers to genes in the other species . These mappings allow cross-species mapping between all homologous gene clusters for currently 20 species. HomoloGene uses RefSeq protein identifiers (NP_, XP_) which are internally mapped to RefSeq genomic identifiers (NM_, XM_). The cross species mapping option is therefore currently only available when genomic RefSeq identifiers are given as input.

### Statistical analysis

The disadvantage of many statistical tests is that they are only applicable if the tested random variable is Gaussian. Many of the gene or promoter properties which are stored in the annotation database, however; are not normally distributed. To obtain unbiased statistical tests, we implemented both, non parametric tests (Kolmogorov-Smirnov) and randomization/bootstrap tests of the mean. ContDist applies three different sets of statistical tests depending on the type of user input and additionally calculates basic statistical parameters estimated from the input samples (Figure 1).

The Kolmogorov -Smirnov test can be applied in the same way for the first two input options (corresponding to Figure 1 A:I-II and B:I-II), however; the randomization/bootstrap tests change slightly between the different options as explained in materials and methods. For the third option where two lists of homologous genes are compared (Figure 1A: III) a paired t-test is used as described in detail in the methods section.

### Case studies

**Figure 2:**
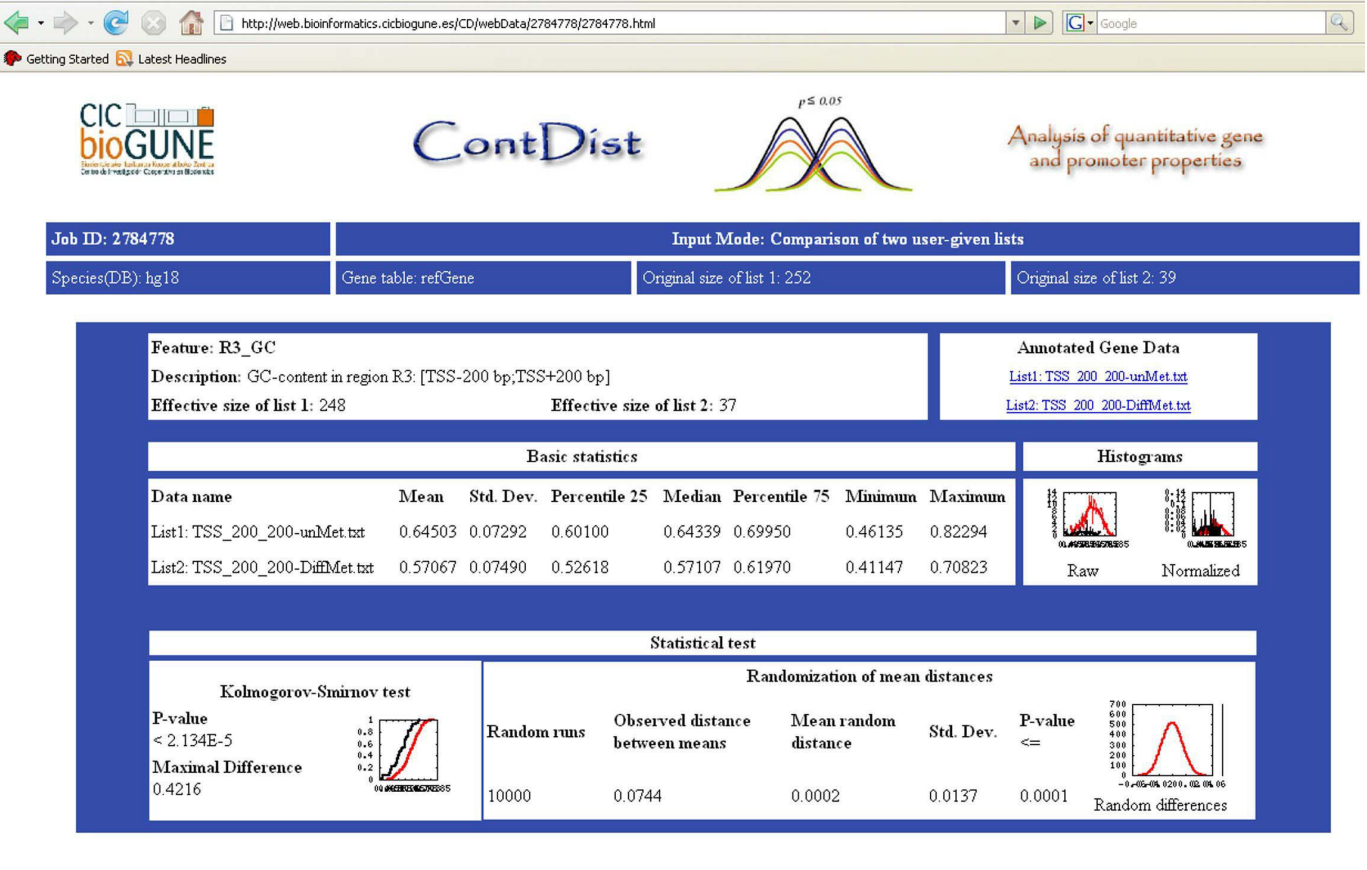
**Graphical display of a typical outcome for the comparison of two gene lists**. The head of the page shows a short summary of the analysis (analysis type, Job ID, input data, data sizes etc.). After the header, an output box is given for each annotation. Each box consists of three different tables: summary, basic statistics of the input and statistical tests. The summary table provides the number of genes for which the chosen annotation exists (effective sizes) and the annotated input data for download. It can be seen that 248 out of 252 and 37 out of 39 genes could be found in the database (differences between original and effective input size). The basic statistic table gives a rough overview on the input data and displays parameters such as means, medians and standard deviation apart of a graphical visualization so that the user can rapidly gain insight on the distribution of the quantitative feature annotated to the input genes. Finally, the last table resumes the statistical tests. In the case of comparing two input lists, two tests are carried out: the Kolmogorov-Smirnov test and a randomization test of the means (see "Randomization/bootstrap statistical tests" in "Materials and Methods"). For both tests, apart from the p-values, the most important values (maximal difference, observed distance between means etc.) and a graphical representation are given.

ContDist is a user friendly tool which dynamically generates the input pages depending on the previous steps. The ContDist webpage contains a step-by-step tutorial . The input procedure is divided into three steps: 1) choose the species from which the input data is derived, 2) choose the gene table and the analysis type (input list vs. reference genes, comparison of two lists and comparison to homologous genes), and 3) select the annotations for analysis (in theory all available annotations can be run simultaneously). Figure 2 shows an output page for a comparison of two input lists. The output pages differ for the different analyses and are explained in more detail on the tutorial page.

The main purpose of this section is to illustrate the general functionality of the tool and its usefulness. As mentioned above, the tool has a strong focus on the promoter regions, and therefore many cases can be conceived in which this tool might deliver important information. For example, for differentially expressed genes it might provide valuable additional information for characterization of the promoter properties of these genes. Furthermore, this tool will help to characterize and further analyze genes obtained by many of the emerging high-throughput techniques (an example of such a gene list is given in the first case study). Finally, this tool can be used to carry out *in silico *research which we demonstrate by means of the second case study; the analysis of housekeeping vs. tissue specific genes.

#### Case study 1: Genes with unmethylated vs. differentially methylated promoter

As mentioned above, the tool has a strong focus on the promoter regions. In the first case study we therefore used two gene lists derived from the methylation states of the promoter regions. For this aim we used the data from the Human Epigenome Project (HEP) which recently released methylation data of approx. 1.9 million CpGs over 12 tissues in the chromosomes 6, 20 and 22 [29]. From this data we obtained two gene lists: one with unmethylated promoter regions (hypomethylated promoters) and another one with differentially methylated promoter regions (see "Test data sets for case study" in "Materials and Methods").

Table 2 presents a summary of the statistical comparisons we carried out with ContDist for these two gene lists. The annotations in table 2 were selected since they allow not only the confirmation of recently reported findings but also the detection of new discoveries.

**Table 2:** A summary of the comparison of unmethylated and differentially methylated promoters.

	**UnM**		**Differentially M**			
Feature	Mean	Median	Mean	Median	p-value_KS_	p-value_Rand_

G+C in R3	***64.50***	***64.34***	***57.07***	***57.11***	***2.13E-05***	***1.00E-04***
G+C in R6	50.04	49.87	49.02	47.87	4.70E-01	0.3731
G+C in introns	47.31	47.3	49.55	49.58	6.35E-01	0.1699
G+C in 3' UTR	45.8	46.29	47.17	48.47	5.67E-01	0.4603
G+C in 3 position	60.91	63.42	64.37	69.08	3.10E-01	0.2065
CA density in R3	***0.0523***	***0.05***	***0.06493***	***0.065***	***3.37E-05***	***1.00E-04***
CG density in R3	***0.08597***	***0.085***	***0.04297***	***0.0375***	***1.53E-09***	***1.00E-04***
TG density in R3	***0.06039***	***0.06***	***0.07723***	***0.0775***	***3.31E-06***	***1.00E-04***
Twist in R3	***0.02515***	***0.02518***	***0.02564***	***0.0257***	***4.91E-10***	***1.00E-04***
Tilt in R3	0.03556	0.03556	0.03546	0.03548	3.29E-01	0.1409
Rise in R3	***7.82035***	***7.82304***	***7.79215***	***7.79555***	***2.79E-07***	***1.00E-04***
Roll in R3	***0.01984***	***0.01984***	***0.01979***	***0.01979***	***3.19E-04***	***1.00E-04***
Shift in R3	***1.31184***	***1.31192***	***1.32067***	***1.32122***	***3.08E-03***	***0.0004***
Slide in R3	***2.09794***	***2.09697***	***2.11725***	***2.1185***	***1.16E-02***	***0.0014***
Bock-comb in R1	***0.59871***	***0.5875***	***0.43191***	***0.415***	***5.67E-05***	***1.00E-04***
unMeth prob in R3	***0.65596***	***0.79195***	***0.3217***	***0.20742***	***4.26E-08***	***1.00E-04***
Ka/Ks hsa/mmu	***0.10729***	***0.08677***	***0.20001***	***0.15901***	***8.09E-04***	***0.0003***
nucC hsa/mmu	***0.14416***	***0.13743***	***0.21186***	***0.19773***	***1.68E-05***	***1.00E-04***
protC hsa/mmu	***0.11891***	***0.09942***	***0.24044***	***0.22569***	***4.63E-05***	***1.00E-04***
Nc	48.29	49.26	47.03	47.82	6.38E-01	0.3138
peakExpression	3727	1392	2966	1403	6.77E-01	0.6982
Expression Breadth	***66.00***	***81.65***	***51.30***	***50.63***	***4.07E-02***	***0.0234***

Significant differences are highlighted in bold. "R" is used to specify different promoter regions: R1 is the Transcription Start Site (TSS), R3 [TSS -200 bp; TSS + 200 bp] and R6 [TSS -1500 bp; TSS]. G+C stands for the GC-content, Bock-comb is the combinatorial score (see ) of Bock CpG islands overlapping the TSS (R1 regions) and "unMeth prob in R3" is the mean probability of the R3 region to remain unmethylated. nucC and protC are the substitution probabilities per base/amino acid (hsa/mmu indicates that the values are based on pair wise alignments between human and mouse) and Nc is the codon bias. M is the abbreviation of "methylated".

In first place we analysed the differences in GC-content between the two gene lists in several gene regions. We found the differences to be highly significant in the R3 region (symmetric region of 200 bp around the TSS). Promoters which are generally unmethylated are by far GC-richer than those which show differential methylation (64.5% vs. 57%). Interestingly, this difference vanishes when analyzing a larger upstream region R6 (TSS -1500 bp; TSS) and also the GC-content of the introns does not show a significant difference between the two gene lists. This suggest firstly, that the difference in GC-content is highly concentrated in a short region around the TSS [30] and secondly, that these differences are not due to the location in different isochores but real differences in the promoter type. Furthermore, another indicator of isochore membership, the GC-content in the third position of the codons [31], seems to confirm this as it does not show a significant difference between the two gene lists.

Recently it has been shown that strong CpG promoters (high CG density) are mostly unmethylated even when inactive while CpG poor promoters seem to be the preferential targets for *de novo *methylation in somatic cells [18]. Our results in table 2 seem to confirm this finding. The mean CpG density in R3 promoter regions of unmethylated genes is twice as high as the corresponding density in differentially methylated promoters (P_KS _< = 1.53E-09, P_random _< 0.0001). Methylated CpGs are prone to mutate towards TG/CA, and table 2 shows that the densities of these dinucleotides are higher in differentially methylated promoters. This indeed suggests higher *de novo *methylation and evolutionary loss of CpGs (substitutions towards TG/CA).

Next, we analyzed the differences in DNA properties like bending and curvature (helical deformations) which to our knowledge have not been analyzed in this context. Table 2 shows that a large difference exists in the Twist (rotation around the center line of DNA helix). This means that differentially methylated promoters are significantly stiffer than unmethylated promoters under this rotation. On the contrary, another rotational deformation, Tilt, does not show a significant difference between the two methylation gene lists. This is interesting as it means that a difference in GC-content does not necessarily implies a difference in the mean DNA properties.

Two predictions on methylation probabilities and chromatin states were also analyzed. The mean probability to remain unmethylated is 0.66 in the unmethylated gene list while it is just half as much, 0.32 in the differentially methylated promoters. The epigenetic scores given by Bock *et al*. [17] can range from 0 to 1: 0 indicates completely silenced and inaccessible regions and 1 means completely unmethylated and highly accessible regions. Table 2 shows additionally that the epigenetic score indicate also less methylation and a more open chromatin structure for the unmethylated promoters.

Another interesting outcome is related to the conservation of the genes. The results indicate that the genes with unmethylated promoters are more conserved (the coding region) than genes with differentially methylated promoters. This applies to both the substitution probability per base and amino acids and it is also reflected in the Ka/Ks ratios. Values near 0 indicate strong negative selection while values higher than one might point out positive selection [19]. The mean Ka/Ks ratio of unmethylated promoters (0.107) is just half as high as in the case of differentially methylated promoters (0.200).

Finally, we found that neither the peak expression (value for the tissue with the highest expression) nor the mean expression (mean expression over all tissues) show significant differences between the two gene lists. However, the expression breadth (% of tissues where the gene is expressed) shows a significant difference (66% in unmethylated genes vs. 51.3% in differentially methylated genes). It is known that approx. 60% of all genes posses a CpG island overlapping its TSS and that CpG islands are higher correlated with housekeeping genes than with tissue specific genes [32]. Given the higher CpG density observed in unmethylated promoters, it can be assumed that they are also more correlated to CpG islands. However, a higher correlation towards CpG islands implies also a higher correlation to housekeeping genes, what in turn can explain the higher observed expression breath in unmethylated genes.

#### Case study 2: Housekeeping (HK) vs. tissue specific genes (TS)

The lists of housekeeping and tissue specific genes where derived from the expression data described above in the section 'Gene Expression'. We considered a gene as housekeeping if it is expressed in all 79 tissues (including pathologic tissues). On the other hand, we define the tissue specific genes to have an expression breadth lower than 10% (e.g. expressed in less than 8 tissues).

The differences between housekeeping and tissue specific genes have been intensively studied over the last years (for a recent update, see [33] and references therein). The understanding of the differences between these two groups regarding its genomic structure, evolutionary rate and transcriptional regulation is fundamental to understand transcriptomics in general. We have chosen this example to demonstrate that the presented tool can also be used to carry out *in silico *research.

Several well established differences do exist between HK and TS genes, like the higher expression rates, the high association of HK genes with CpG islands and the higher conservation of the coding region in HK genes. In table 3 we confirm these well established differences between these two groups, showing in this way that by means of the presented tool many fundamental gene properties can be analyzed and compared in an easy and quick way. No consensus does exist on the lengths of the coding regions and mRNAs. The results shown in table 3 coincide with studies based on microarray expression data (like the input data used in this case study) showing that TS genes seem to have slightly longer CDS than HK genes. Finally we also analyzed the differences in the number of protein-protein interactions. We found a significant difference between the two gene groups showing the HK genes have more PPI than the TS genes, a result which to our knowledge has not been reported before.

**Table 3:** Basic differences between housekeeping and tissue specific genes.

	**housekeeping**		**tissue specific**				
Feature	Mean	median	mean	median	ratio	p-value_KS_	p-value_Rand_
CG density in R3	***0.086***	***0.088***	***0.064***	***0.060***	***0.416***	***7.16E-80***	***0.0001***
G+C in R2	***65.6***	***66.7***	***61.8***	***62.7***	***0.085***	***1.83E-27***	***0.0001***
G+C in R3	***64.6***	***65.6***	***60.9***	***62.3***	***0.086***	***1.06E-25***	***0.0001***
G+C in R4	***58.7***	***58.8***	***56.3***	***56.9***	***0.061***	***1.66E-17***	***0.0001***
G+C in R6	***51.7***	***51.3***	***50.6***	***50.4***	***0.033***	***1.31E-17***	***0.0001***
G+C in Intron	46.6	45.6	47.1	45.8	-0.016	5.22E-06	0.0514
Ka/Ks (hsa/mmu)	***0.100***	***0.074***	***0.151***	***0.125***	**-*0.594***	***8.25E-35***	***0.0001***
Subst. per aa (hsa/mmu)	***0.107***	***0.082***	***0.171***	***0.147***	**-*0.675***	***2.76E-47***	***0.0001***
PPI	***7.9***	***3.0***	***4.8***	***2.0***	***0.728***	***7.28E-10***	***0.0004***
mean Expr.	***2264.1***	***1137.7***	***933.4***	***287.3***	***1.278***	***0.00E+00***	***0.0001***
peak Expr.	***9716.4***	***3660.3***	***2229.0***	***357.8***	***2.124***	***0.00E+00***	***0.0001***
CDS length	***1532.6***	***1170.0***	***1703.0***	***1335.0***	**-*0.152***	***2.34E-10***	***0.0001***
mRNA length	2900.4	2439.5	2964.7	2427.0	-0.032	9.48E-01	0.2685

The tool permits to confirm quickly some known aspects like the difference in expression levels, association to CpG islands and higher conservation of HK genes. The tool also detects that HK genes products have on average more interaction partners, a finding which to our knowledge has not been reported so far.

## Conclusion

We present ContDist, a tool which can analyze and compare continuously distributed gene and promoter properties. Thus, the novelty of our tool resides in its strong focus on the promoter regions, which have been widely disregarded so far by others, and the fact that ContDist uses quantitative features instead of labelled annotations (like in "traditionally" ontological analyses). Currently, the annotation database holds around 200 different gene and promoter features for each of the 7 species which are currently available. It implements 3 different analysis options: comparison of two input gene lists, a gene list vs. a reference list and the comparison of an input list with its corresponding homologous genes. For each of the analysis types, the appropriate statistical tests are implemented like the Kolmogorov-Smirnov test, randomization tests of the mean or paired t-test. The output displays in a concise way the statistical significances, graphical representation of both the annotated input data and the statistical tests and a basic statistics of the input data. Moreover, it also provides the annotated gene lists for download. Therefore, this tool can also be used for annotation purposes.

We showed the usefulness of the tool by means of two case studies. In the first one, we compared genes with unmethylated to those with differentially methylated promoters and in the second one we analysed the fundamental differences between housekeeping and tissue specific genes. The latter also demonstrates the usefulness of this tool in fundamental *in silico *studies. In both case studies we could quickly confirm some recent findings like the increased probability of *de-novo *methylation for less CpG-dens promoters [18] or the higher expression levels of HG genes. However, we also demonstrated that ContDist can reveal new insight to biology function. We reported striking differences in the mean stiffness of different helical deformations of DNA between unmethylated and differentially methylated promoters. It turned out that the rotation around the center line of the DNA helix (Twist) is much stiffer in differentially methylated promoters. On the contrary, no significant difference could be found for another rotational deformation (Tilt) which shows that base composition cannot account for the differences found for Twist. Finally, the tool also revealed that genes with unmethylated promoters are much more conserved than genes with differentially methylated promoters and a significant difference in the number of protein-protein interactions between housekeeping and tissue specific genes.

## Methods

### Software implementation

The ContDist user interface was written in php which allows the dynamic generation of the HTML input pages depending on previously provided parameters. For example, the chosen specie will affect the later options available. The core algorithms of ContDist are implemented in Java. The Java algorithms communicate with the MySQL database and perform the appropriate statistical tests. ContDist interfaces also to GNU plot to generate histograms, cumulative fraction plots, and other statistical plots.

### Randomization/bootstrap statistical tests

Randomization/bootstrap statistical tests are applied when comparing an input list to a reference gene list and/or comparing two independent gene lists. However, the test is carried out in a different way depending on which of the two user-given input options is selected. Note that the first test can be compared to a one sample t-test (null hypothesis: the input gene list is randomly extracted from the reference genes) while the second randomization test would correspond to a two sample t-test (null hypothesis: the genes are randomly assigned to the lists).

1. In the first case where an input list is compared against a reference gene list, the input list is a subset of the reference genes (for the analysis the input genes are not removed from the reference genes). To establish if the mean value of the input list is significantly different to the mean value of the reference genes, a sampling distribution of the reference set is generated. 10000 random lists are sampled out from the reference with the same size as the input list and the mean value is calculated for each random sample. The resulting distribution of randomly generated mean values (sampling distribution) will be normally distributed if the input gene list is sufficiently large. The standard z-score can now be calculated as: $z=\frac{x-\mu }{\sigma }$ where μ is the mean of the population (randomizations) and σ the standard deviation of all random runs. x is the value to be standardized. The z-score is the number of standard deviations an observed value is away from the population mean (the mean value of all randomizations). The corresponding p-value can be calculated easily. First, the p-value for a one-sided test is calculated by means of the cumulative density function of the Gaussian distribution as 1-CDF_x _(x denotes the value of the z-score). Finally, applying the doubling approach (multiplying by 2) we obtain the p-value for a two-tailed test.

In addition, a bootstrap p-value is calculated by counting the number of times the random mean is smaller or higher than the observed mean (mean of the input list). Then we define the bootstrap p-value as:

(1) $$p-value_{bootstrap}=\frac{2\cdot \min\{N_{higher},N_{lower}\}}{N}$$

with

(2) $$\begin{array}{ccc} N_{higher}=\sum_{i=1}^{N}\delta_{i} & with & \delta_{i}=\{\begin{array}{c} 1:x_{i}\geq x\\ 0:rest \end{array} \end{array}$$

and N_lower _= N - N_higher _being N the number of randomizations.

2. For the case where two independent gene lists are compared, a randomization test of the means [34] is performed in order to establish if the quantitative values in the two input lists are significantly different. First, the difference between the means of the two lists is calculated which we will refer to as the observed distance. The values are then randomly reassigned 10000 times to the two lists maintaining the original sizes of the lists. For each random run the difference between the two random lists are calculated. This gives a (re)-sampling distribution of the differences. The p-value of the randomization test can now be calculated as:

(3) $$p\leq \frac{N_{higher}^{abs}}{N}$$

N_higher _is the number of random differences which are higher or equal to the observed difference. The null hypothesis is that the two lists are equal which means that a two-tailed p-value is needed. This is achieved by using the absolute in the Kronecker delta function x_i _> = |x| (in equation 2)

### Paired t-test statistical test

For the case where two lists of homologous genes are compared the values of both lists are correlated (e.g. the two values of a particular pair of homologous genes correspond to the same gene in different species) and subsequently the gene lists are considered dependent. This fact should be taken into account when performing a statistical test. Therefore, in order to test if the two distributions have equal means, a paired t-test is required. This test can be applied if the distribution of the differences between the two lists is Gaussian. ContDist plot the distribution of differences, so that the user can easily check if this criterion holds.

### Test data sets for case study

The methylation data from the HEP home page  was downloaded and used as test case for ContDist. Currently, methylation data exists for 12 tissues in 3 chromosomes (6, 20 and 22). All valid CpGs, together with the information on methylation values and the tissue is mapped to the promoter region of all genes comprised by TSS -200 bp to TSS + 200 bp. In the next step the mean percentage of promoter methylation is calculated for every promoter region and for all tissues (the methylation values reach from 0, e.g. unmethylated, to 100 corresponding to fully methylated). We just consider promoters with at least 4 CpGs mapped in 4 different tissues. We define a promoter as unmethylated if the mean methylation is smaller than 20 in all tissues (a similar definition can be found in [29]). Consequently, we consider a promoter as methylated if the mean methylation is higher than 80 in all tissues. Promoters with intermediate methylation (>20 and <80) were not considered. Finally, we define a promoter as differentially methylated if it is in at least one tissue methylated and in one tissue unmethylated. In the RefSeq gene table, many transcripts of splice variants are annotated which start at the same position and this may lead to duplicated promoters in the analysis. Therefore, we filtered out redundant promoters for this specific test case by means of grouping the genes by their TSS maintaining finally just one gene of each TSS group (filtering duplicated promoters). This procedure yields a data set consisting of 252 unmethylated and 39 differentially methylated promoters.

## Availability and requirements

The web tool is available under . It can be freely accessed and no login is required.

## Authors' contributions

RM and MH coordinated the work, MH formulated the main idea, designed and populated the database, programmed the core algorithms and wrote the manuscript, GL was involved in the design and programming of the user interface and commented the manuscript; RM supervised the work and edited the manuscript.
